# True dorsalis pedis artery aneurysm: case report

**DOI:** 10.1590/1677-5449.012817

**Published:** 2018

**Authors:** Uellinton Galli Ferreira, José Aderval Aragão, Antonio Alexandre Lenik, Iapunira Catarina Sant’Anna Aragão, Felipe Matheus Sant’Anna Aragão, Wilson Barbosa Leão, Carlos Eduardo Nunes, Francisco Prado Reis

**Affiliations:** 1 Fundação Beneficência Hospital de Cirurgia, Serviço de Cirurgia Vascular Dr. José Calumby Filho, Aracaju, SE, Brasil.; 2 Universidade Federal de Sergipe – UFS, Aracaju, SE, Brasil.; 3 Universidade Tiradentes – UNIT, Aracaju, SE, Brasil.; 4 Centro Universitário de Volta Redonda – UNIFOA, Volta Redonda, RJ, Brasil.

**Keywords:** aneurysm, artery, systemic arterial hypertension, vascular surgical procedures

## Abstract

A true aneurysm of the dorsal artery of the foot is a rare medical finding and its principal causes and clinical manifestations are not well known. A 49-year-old female patient presented with a pulsatile mass on the dorsal part of her right foot. Clinical and ultrasound examinations confirmed a diagnosis of aneurysm. The aneurysm was resected after dissection, exposure and isolation of the proximal and distal stumps of the dorsalis pedis artery. Simple ligature and resection of the aneurysm is proving to be a safe treatment option in patients with a patent plantar arch.

## INTRODUCTION

 Aneurysms of the dorsal artery of the foot are extremely rare and their clinical manifestations are not well known. 1 ^,^ 2 Descriptions available in the literature are contained in case reports 3 and, since the first description published in 1907 by Cauff, 4 a variety of different treatment methods have been proposed. 5 ^,^ 6 However, the majority of dorsalis pedis aneurysms are pseudoaneurysms secondary to traumas. 3 ^,^ 7

## CASE REPORT

 A 49-year-old female patient described a pulsating mass on the dorsal aspect of the right foot with onset approximately 3 years earlier that had grown progressively before becoming painful a few months prior to presentation, which caused her to seek medical care. She stated that she had not suffered any traumas or undergone any surgical procedures to the foot, had no family history of aneurysms, diabetes, or dyslipidemia, but was a smoker and had hypertension as cardiovascular risk factors. 

**Figure 1:**
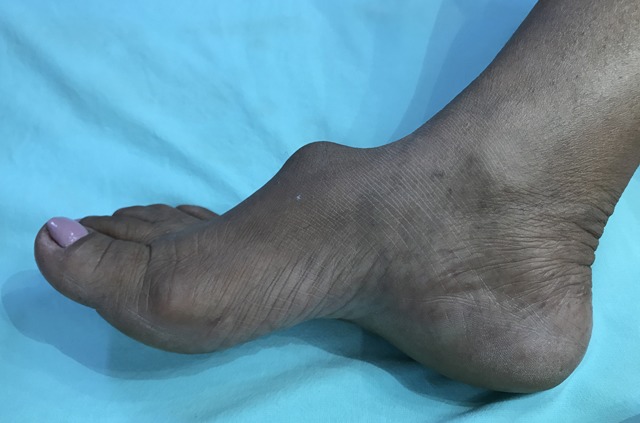
Compressible, painful, pulsating mass on the dorsal aspect of the foot.

 On physical examination, a pulsating mass, static and painful on palpation, was observed on the dorsal aspect of the right foot, suggestive of an aneurysm of the dorsal artery of the foot ( Figure 1 ). Additionally, there was a strong pulse in the posterior tibial artery, with no signs of chronic ischemia or other detectable vascular disorders. 

**Figure 2:**
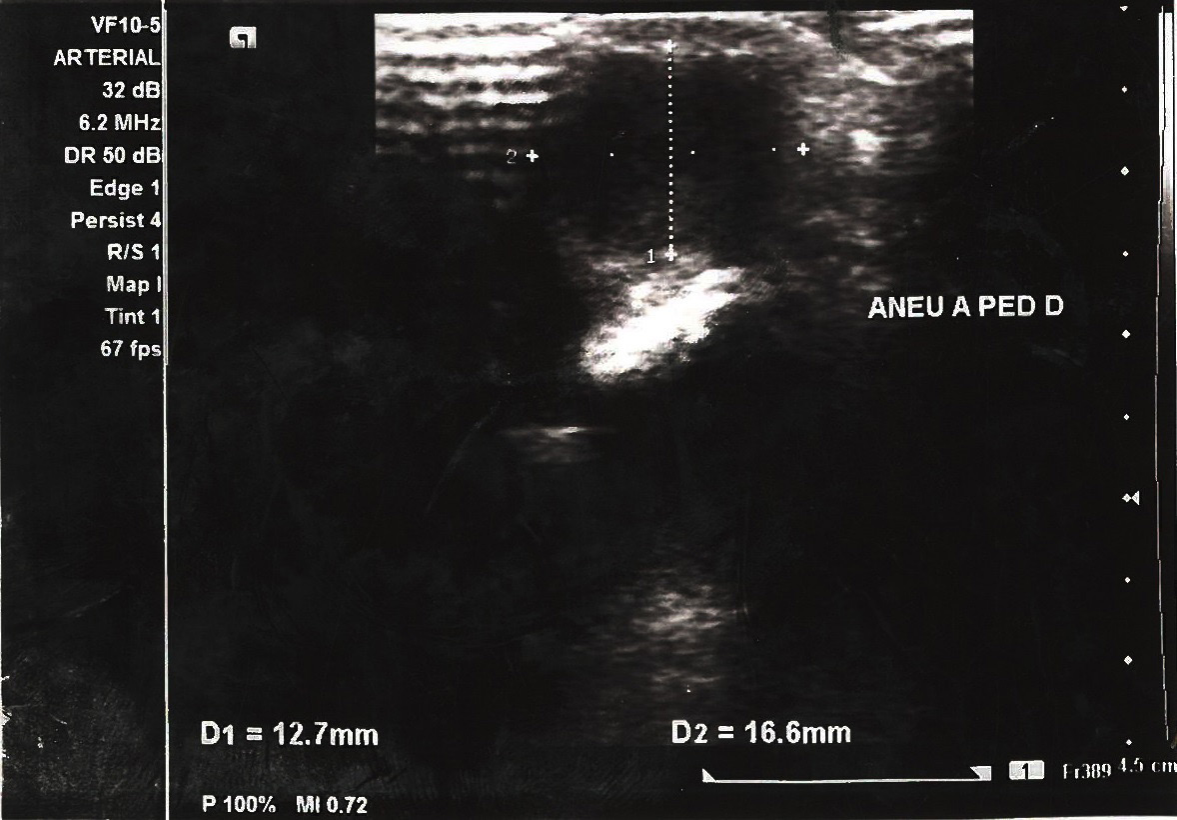
Aneurysmal dilatation of the dorsal artery of the right foot seen on ultrasound.

 Ultrasonography showed an oval, anechoic image along the course of the dorsal artery of the right foot, measuring approximately 1.2 × 1.6 × 2.2 cm ( Figure 2 ). 

**Figure 3:**
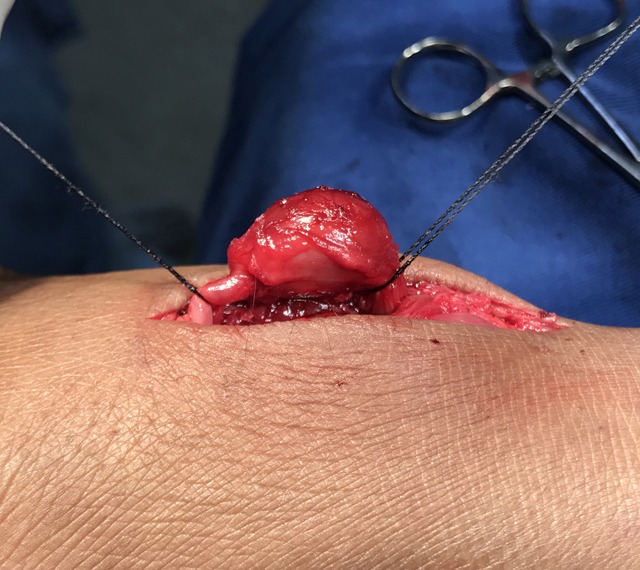
Surgical exposure of a saccular aneurysm of the dorsal artery of the right foot with proximal and distal stumps repaired.

 Exploratory surgery, under local anesthesia, was initiated with a longitudinal incision in the dorsal surface of the right foot, above the aneurysm. After careful and detailed dissection, a dilation with a saccular appearance was observed along the course of the dorsal artery of the foot. After exposure, the proximal and distal stumps of the dorsal artery of the foot were isolated and ligated and the aneurysm was resected ( Figure 3 ). 

**Figure 4:**
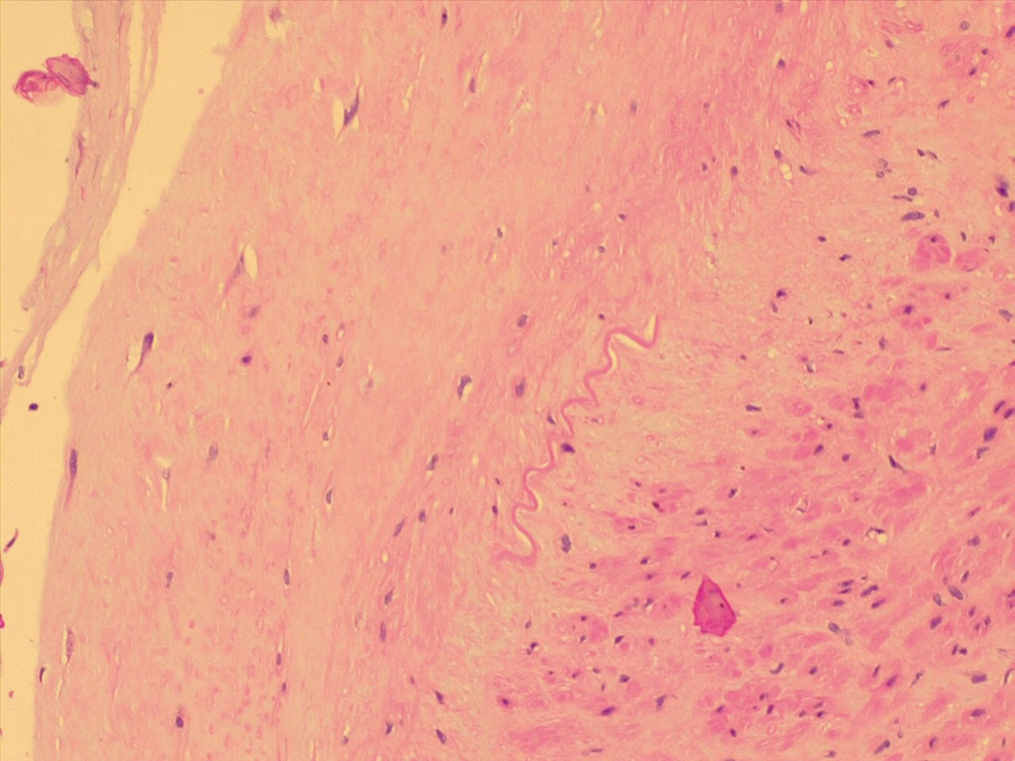
Histopathological study of the dorsal artery of the right foot, showing intimal thickening and myxoid degeneration of the aneurysm sac.

 Reconstruction of the artery was considered unnecessary, since the foot showed no signs of ischemia and duplex scanning revealed excellent flow to the interdigital and tibial arteries. Histopathological analysis of the aneurysm sac found intimal thickening and myxoid degeneration with inflammatory infiltrate and atherosclerotic changes ( Figure 4 ). 

## DISCUSSION

 True aneurysms of the dorsalis pedis artery are rare and their pathophysiology has not been elucidated. 5 ^,^ 8 According to Aragão et al., 1 just 24 cases have been described in the literature to date. However, the majority of aneurysms involving infrapopliteal arteries are generally of traumatic origin. 2 ^,^ 9 It is important to rule out traumas or procedures affecting the lower limbs, since the majority of cases of aneurysms of the dorsal artery of the foot are actually pseudoaneurysms rather than true aneurysms. 1 ^,^ 5 ^,^ 6

 According to Legel et al., 3 Al-Omran, 10 and Kwon et al., 11 the major causes of pseudoaneurysms are venipuncture to draw blood, local traumas, orthopedic surgery, and vascular surgery. However, true aneurysms of the dorsal artery of the foot are very often associated with arterial hypertension, diabetes, smoking, and atherosclerosis. 1 ^,^ 5 ^,^ 9 In our case both smoking and hypertension were present. 

 The most frequently described clinical manifestations of aneurysms of the dorsal artery of the foot are a pulsating mass, painful or painless, sometimes associated with itching and discomfort. 5 ^,^ 7 In the case described here, the aneurysm was asymptomatic at onset and only began to cause pain and discomfort after progressive increase in the size of the pulsating mass on the dorsal aspect of the right foot, which may have been caused by compression of adjacent structures. The majority of patients with aneurysms of the dorsalis pedis artery were male (63%) and mean age was 55 years, 2 in contrast with our patient who was female and 49 years old. 

 Several authors have proposed different methods to treat aneurysms of the dorsal artery of the foot, such as resection and simple ligature and revascularization with end-to-end anastomosis or saphenous vein interposition. 1 ^,^ 5 ^,^ 9 ^,^ 12 ^-^ 15 In our case, we chose simple ligature and resection of the aneurysm sac because the limb showed no clinical signs of ischemia. However, patients at elevated risk of peripheral vascular diseases or diabetes and children may benefit from revascularization to avoid future complications, such as ischemia, necrosis, and limb loss. 

## CONCLUSIONS

 True aneurysms of the dorsal artery of the foot are extremely rare. Simple ligature of the dorsalis pedis artery and resection of the aneurysm are proving to be a simple and safe treatment when the plantar arch is patent and the foot shows no chronic signs of ischemia. However, revascularization is recommended for patients with vascular risk factors and peripheral arterial disease, to avoid possible complications and amputation. 
