# Supplementary material for: True dorsalis pedis artery aneurysm: case report
Source: J Vasc Bras. 2018 Apr-Jun;17(2):152–5. doi: 10.1590/1677-5449.012817 (PMC6205713; doi:10.1590/1677-5449.012817)
Supplement: Translation

# Translation: Aneurisma verdadeiro de artéria dorsal do pé: relato de caso

**Authors:** Uellinton Galli Ferreira, José Aderval Aragão, Antonio Alexandre Lenik, Iapunira Catarina Sant’Anna Aragão, Felipe Matheus Sant’Anna Aragão, Wilson Barbosa Leão, Carlos Eduardo Nunes, Francisco Prado Reis

**Affiliations:** 1 Fundação Beneficência Hospital de Cirurgia, Serviço de Cirurgia Vascular Dr. José Calumby Filho, Aracaju, SE, Brasil.; 2 Universidade Federal de Sergipe – UFS, Aracaju, SE, Brasil.; 3 Universidade Tiradentes – UNIT, Aracaju, SE, Brasil.; 4 Centro Universitário de Volta Redonda – UNIFOA, Volta Redonda, RJ, Brasil.

**Keywords:** aneurisma, artéria, hipertensão arterial sistêmica, procedimentos cirúrgicos vasculares

## Abstract

O aneurisma verdadeiro da artéria dorsal do pé é um achado médico raro, e suas principais causas e manifestações clinicas são pouco conhecidas. Paciente do sexo feminino, 49 anos de idade, apresentou-se com uma massa pulsátil no dorso do pé direito. O exame clínico e a imagem ultrassonográfica confirmaram o diagnóstico de um aneurisma. O aneurisma foi ressecado após dissecação, exposição e isolamento dos cotos proximal e distal da artéria dorsal do pé. A ligadura simples e a ressecção do aneurisma vêm se mostrando um tratamento seguro quando o arco plantar se mantém pérvio.

## INTRODUÇÃO

 O aneurisma de artéria dorsal do pé é extremamente raro e suas manifestações clinicas pouco conhecidas 1 ^,^ 2 . Tem sido reportado na literatura através de relatos de caso 3 , e desde a primeira descrição por Cauff 4 em 1907 foram propostas diferentes formas de tratamento 5 ^,^ 6 . Entretanto, a maioria dos aneurismas de artéria dorsal do pé são pseudoaneurismas secundários a traumas 3 ^,^ 7 . 

## RELATO DE CASO

 Paciente de 49 anos, sexo feminino, relata aparecimento de uma massa pulsátil no dorso do pé direito há aproximadamente 3 anos, apresentando crescimento progressivo e dor no decorrer dos últimos meses, o que levou a paciente a procurar assistência médica. Nega traumas ou procedimentos cirúrgicos no pé, antecedentes familiares de aneurismas, diabetes ou dislipidemia, entretanto apresenta tabagismo e hipertensão como fatores de risco cardiovascular. 

 Ao exame físico, foi observada uma massa pulsátil, fixa, dolorosa á palpação, no dorso do pé direito, sugestiva de aneurisma de artéria dorsal do pé ( Figura 1 ). Além disso, observou-se pulso amplo em artéria tibial posterior, sem sinais de isquemia crônica ou outras alterações vasculares detectáveis. 

**Figura 1:** Massa pulsátil, compressível e dolorosa sobre o dorso do pé direito.

 Na ultrassonografia foi evidenciada uma imagem ovalada, anecoica, situada em continuidade com a artéria dorsal do pé direito, medindo aproximadamente 1,2 × 1,6 × 2,2 cm ( Figura 2 ). 

**Figura 2:** Dilatação aneurismática da artéria dorsal do pé direito visto pelo ultrassom.

 A exploração cirúrgica, sob anestesia local, foi realizada através de uma incisão longitudinal no dorso do pé direito sobre o aneurisma. Após disseção minuciosa, foi possível identificar uma dilatação de aspecto sacular em continuidade com artéria dorsal do pé. Após exposição, os cotos proximal e distal da artéria dorsal do pé foram isolados, ligados e o aneurisma ressecado ( Figura 3 ). 

**Figura 3:** Exposição cirúrgica de um aneurisma sacular da artéria dorsal do pé direito com coto proximal e distal reparados.

 A reconstrução arterial não foi necessária, visto que o pé não apresentava sinais de isquemia e foi verificado excelente fluxo para as artérias interdigitais e tibiais através do *duplex scan*. A análise histopatológica do saco aneurismático apresentou espessamento intimal e degeneração mixoide com infiltrado inflamatório e alterações ateroscleróticas ( Figura 4 ). 

**Figura 4:** Estudo histopatológico da artéria dorsal do pé direito, mostrando espessamento intimal e degeneração mixoide do saco aneurismático.

## DISCUSSÃO

 O aneurisma verdadeiro da artéria dorsal do pé é incomum e sua fisiopatologia segue ainda obscura 5 ^,^ 8 . Para Aragão et al. 1 , somente 24 casos foram relatados até o momento na literatura. Porém, a maioria dos aneurismas envolvendo artérias infrapoplíteas comumente é de origem traumática 2 ^,^ 9 . Afastar antecedentes de traumas ou procedimentos no membro inferior é importante, uma vez que, na maior parte dos casos de aneurisma descritos na artéria dorsal do pé, tratava-se de pseudoaneurimas e não de aneurismas verdadeiros 1 ^,^ 5 ^,^ 6 . 

 Para Legel et al. 3 , Al-Omran 10 e Kwon et al. 11 , as principais causas de pseudoaneurismas são punções venosas para coleta de sangue, traumas locais, cirurgias ortopédicas e cirurgias vasculares. Porém, o aneurisma verdadeiro da artéria dorsal do pé está muitas vezes associado a hipertensão arterial, diabetes, tabagismo e aterosclerose 1 ^,^ 5 ^,^ 9 . No nosso caso também estavam presentes o tabagismo e a hipertensão. 

 As manifestações clínicas mais descritas em aneurisma de artéria dorsal do pé eram uma massa pulsátil, dolorosa ou não, por vezes associada a prurido e desconforto 5 ^,^ 7 . No presente relato, o aneurisma a princípio era assintomático, e só após o aumento progressivo da massa pulsátil no dorso do pé direito evoluiu com dor e desconforto, o que poderia ser justificado pela compressão de estruturas adjacentes. A maioria dos pacientes com aneurismas da ADP era do sexo masculino (63%) com idade média de 55 anos 2 , o que difere do nosso caso, em que o paciente era do sexo feminino e tinha 49 anos de idade. 

 Vários autores propuseram diferentes formas de tratamento para o aneurisma de artéria dorsal do pé, tais como resseção e ligadura simples, revascularizações com anastomose termino-terminal ou interposição de veia safena 1 ^,^ 5 ^,^ 9 ^,^ 12 ^-^ 15 . No presente relato, optou-se por ligadura simples e ressecção do saco aneurismático, uma vez que o membro não apresentava sinais clínicos de isquemia. Porém, pacientes com risco elevado de doenças vasculares periféricas, diabetes e crianças poderiam se beneficiar da revascularização e evitar complicações futuras, como isquemias, necrose e perda do membro. 

## CONCLUSÃO

 O aneurisma verdadeiro de artéria dorsal do pé é extremamente raro. A ligadura simples da artéria dorsal do pé e ressecção do aneurisma vêm se mostrando um tratamento simples e seguro quando o arco plantar se mantem pérvio e o pé não apresenta sinais crônicos de isquemia. No entanto, em pacientes com fatores de risco vascular e doença arterial periférica, se recomenda revascularização de modo a evitar possíveis complicações e perda do membro.
